# Short Growth Duration in *Brassica napus* L.: Physiological and Ecological Bases, Genetic Regulation, Trait Trade-Offs, and Breeding Perspectives

**DOI:** 10.3390/plants15213337

**Published:** 2026-10-28

**Authors:** Yu Xu, Zhen Huang, Mei Han, Zhi Li, Shijie Yu, Zhi Zhao, Bo Song, Xiangying Chen, Cunmin Qu, Taocui Huang

**Affiliations:** 1 Institute of Oil Crops, Chongqing Academy of Agricultural Sciences, Chongqing 400060, China; 2 College of Agronomy and Biotechnology, Southwest University, Chongqing 400715, China; 3 State Key Laboratory of Crop Stress Biology for Arid Areas, College of Agronomy, Northwest A&F University, Xianyang 712100, China

**Keywords:** *Brassica napus*, short growth duration, flowering time, co-localization, trait trade-offs

## Abstract

Rapeseed (*Brassica napus* L.) is a major edible-oil crop worldwide. Its growing season is increasingly constrained by intensive cropping systems, especially the rice–rapeseed rotations that dominate the Yangtze River basin of China. Short growth duration refers to a shorter interval from sowing to maturity, which allows rapeseed to fit these narrow rotation windows and to expand the use of winter-fallow farmland. Under the same sowing date, early maturity results in a shorter growth duration. Growth duration reflects several developmental stages, yet most studies have focused on flowering time. It only partly predicts maturity and reveals little about trade-offs with yield, quality, or stress resistance. Here, we synthesize the physiological and ecological regulation of short growth duration and the genetic loci controlling successive developmental phases, including vegetative growth, the floral transition, flowering, and silique maturation. A key challenge is to combine short growth duration with high yield, good quality, and stress resistance. We examine genome-wide co-localization of growth-duration loci with loci for these traits and identify shared candidate genes. As an empirical test, we also analyze the phenotypic performance of 99 lines from the early-maturity group of the 2021–2025 Chinese National Winter Rapeseed Regional Trials. Future work should resolve links between maturity and other traits to guide selection for suitable growth duration while preserving better yield, quality, and stress resistance.

## 1. Introduction

*Brassica napus* L. (rapeseed) is the second-largest source of edible oil and accounts for approximately 15% of global vegetable-oil production [1]. It is widely cultivated in Europe, Canada, China, India, and Australia [2]. Particularly in China, the largest consumer and a major producer of rapeseed, rapeseed oil serves as the predominant edible vegetable oil [3]. However, the self-sufficiency rate of domestically produced rapeseed oil remains at only approximately 34% in China. Increasing rapeseed production capacity is an important objective. China mainly grows semi-winter rapeseed (~90%), which is predominantly distributed in the Yangtze River basin [4]. Winter-fallow farmland in this region offers scope for rapeseed expansion. However, the rotation with rice confines the rapeseed growing season to a narrow window between late-season rice harvest and the next rice transplanting [5]. A short growth duration helps rapeseed fit this window. At a common sowing date, it is expressed as early maturity. In some rotations, rice harvest delays rapeseed sowing [6,7]. If later sowing does not delay final maturity, the sowing-to-maturity interval is also shortened.

According to the statistics, there are over 4.27 million hectares of winter-fallow farmland suitable for rapeseed cultivation in the Yangtze River basin. Thereinto, approximately 40% is located in double-cropping rice areas [8]. In addition, early-maturing *B. napus* could replace low-yielding, lower-quality *B. rapa* varieties in China’s 0.67 million hectares of spring rapeseed planting area [9,10]. Beyond expanding cultivation area, early-maturing varieties may reduce exposure to heat-forced ripening in heat-prone environments [11,12]. In addition, early maturity in rapeseed is often associated with a shorter and more compact plant architecture, which favors dense planting and mechanized harvesting [13,14,15,16].

Growth period, the interval from sowing to maturity, is a complex quantitative trait. It is governed by the seeding stage, floral transition, flowering duration, and post-flowering seed filling and maturation [17,18]. Most research has focused on flowering time, which is correlated with maturity but does not fully determine it [19]. The molecular basis of flowering time regulation in rapeseed has been systematically reviewed in recent years [20,21]. However, these reviews have predominantly centered on flowering pathways, lacking a systematic framework for short-duration traits and its trade-offs with other traits. For example, numerous studies have reported significant correlations between flowering time and yield, quality, and stress-resistance traits [22,23,24], but these findings have not been systematically integrated. In this review, we systematically summarize current research progress on the physiological basis, ecological responses, and genetic regulation of short growth duration in *B. napus.* We also examine how variation in growth duration relates to yield, quality, agronomic traits, and stress resistance. We also analyze data of the early-maturity group from the 2021–2025 Chinese national rapeseed regional trials [25,26,27,28,29]. Finally, we propose future research directions, aiming to provide theoretical guidance for breeding short-duration rapeseed varieties that combine earliness with high yield, good quality, and strong stress resistance.

## 2. Physiological Mechanisms and Ecological Response of Short-Growth-Duration Rapeseed

### 2.1. Developmental Characteristics and Physiological Regulation

**Figure 1:**
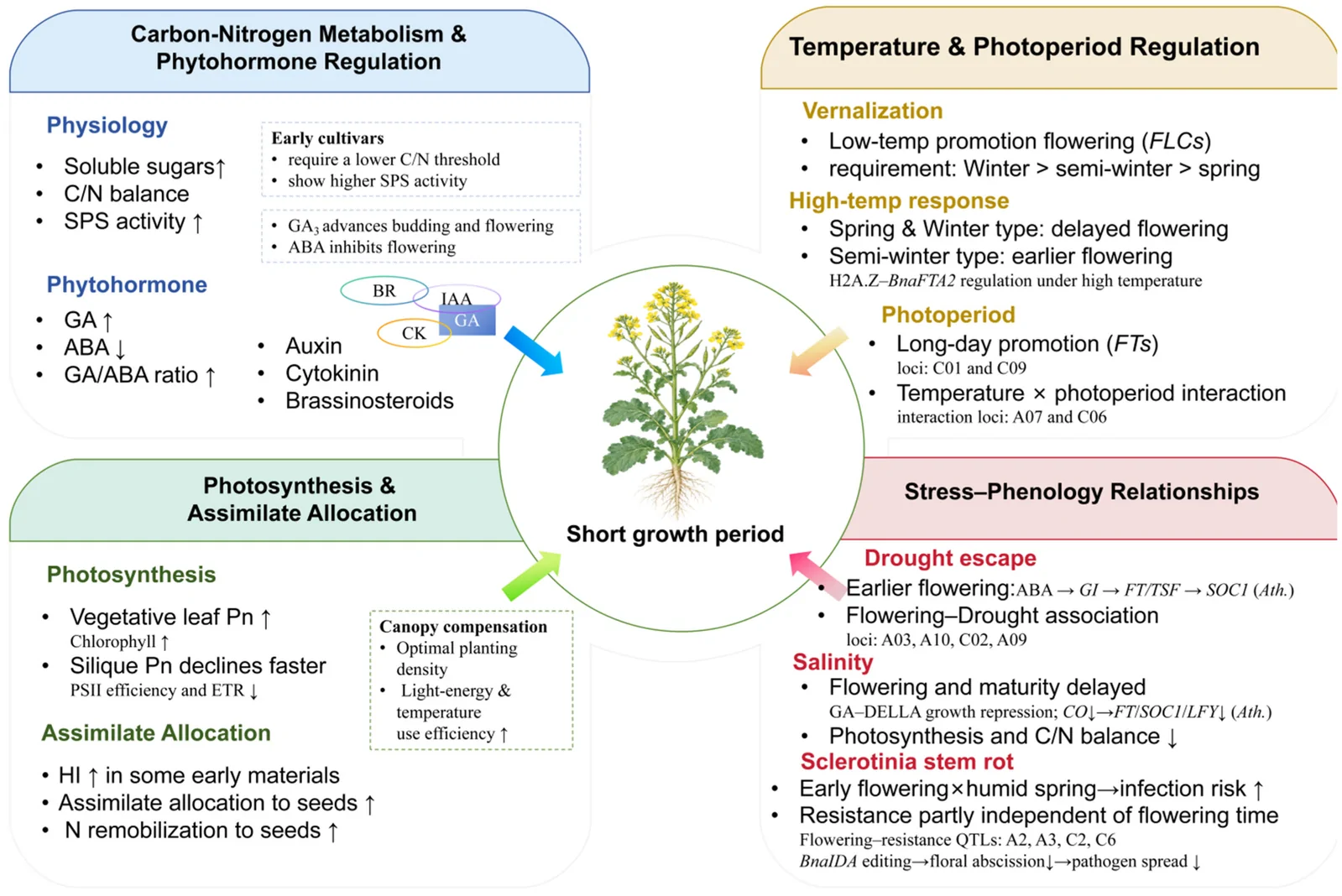
Integrated physiological and ecological regulation of short-growth-period rapeseed. This figure summarizes the physiological and environmental factors that regulate the growth period in rapeseed. The factors covered are carbon–nitrogen metabolism, phytohormones, photosynthesis, assimilate allocation, temperature, photoperiod, and stress. Arrows indicate promotion, and ↑/↓ indicate increases and decreases, respectively. Gene symbols are italicized. ABA, abscisic acid; BR, brassinosteroid; CK, cytokinin; C/N, carbon to nitrogen ratio; ETR, photosynthetic electron transport rate; GA, gibberellin; HI, harvest index; IAA, indole 3 acetic acid; Pn, net photosynthetic rate; PSII, photosystem II; QTL, quantitative trait locus; SPS, sucrose phosphate synthase.

Short-growth-duration rapeseed varieties may differ in the timing and duration of developmental phases. The direction and magnitude of differences in seedling growth, bolting, flowering, and post-flowering development vary among genotypes and environments. Generally, short-growth-duration rapeseed varieties display a shortened seedling phase, accelerated transitions through the budding and flowering stages. Comparative physiological studies have revealed significant differences across rapeseed with different growth duration (Figure 1).

#### 2.1.1. Carbon-Nitrogen Metabolism and Phytohormone Regulation

During floral-bud differentiation, carbohydrate content changes substantially. In *B. napus*, soluble sugars accumulate continuously and peak during flower bud differentiation, while the whole-plant carbon-to-nitrogen (C/N) ratio follows a rise-then-decline trend [30]. From the perspective of growth process, nitrogen metabolism is active before flowering to support vegetative growth. After flowering, carbon metabolism supports reproductive-organ development [31]. These findings indicate that C/N balance may be a key metabolic regulator of flower bud differentiation in rapeseed. Hu et al. reported that a higher C/N ratio promoted floral-primordium differentiation and the transition from vegetative to reproductive growth [32]. A comparison among rapeseed cultivars found a lower C/N threshold at floral-bud differentiation in early-maturing than in later-maturing cultivars, which may partly explain their earlier reproductive transition. In addition, early-maturing cultivars had higher sucrose phosphate synthase (SPS) activity at the initial flowering stage, which potentially supports sucrose production and reproductive growth [33]. However, most current studies remain at the comparison of physiological indicators with different rapeseed varieties. The interactions between C/N metabolism and growth-duration pathways remain unclear.

Phytohormone levels are important physiological factors in developmental progression. Early-maturing cultivars generally accumulate higher gibberellin (GA) and lower abscisic acid (ABA) levels, resulting in a higher GA/ABA ratio than late-maturing cultivars. This is consistent with experiments in which exogenous GA_3_ advanced budding and flowering, whereas ABA inhibited floral development [34]. Auxin and cytokinin (CK) are also involved in floral bud differentiation. Auxin participates in the transition from the shoot apical meristem to the floral meristem, while cytokinin enhances the meristematic activity of the shoot apex. Both can interact with GA to promote budding and flowering [35,36]. Brassinosteroids (BRs) can promote seedling growth through auxin signaling, potentially affecting the duration of the vegetative phase [37]. The low-temperature vernalization process is accompanied by hormonal changes, including reduced ABA and increased GA and CK levels [38,39].

#### 2.1.2. Photosynthetic Characteristics and Assimilate Allocation

Photosynthetic differences between early- and later-maturing rapeseed cultivars vary with developmental stage. During vegetative growth, some early-maturing cultivars often show higher net photosynthetic rates and chlorophyll contents in functional leaves. This helps to accumulate biomass within a shorter vegetative period [40]. However, the net photosynthetic rate of siliques declines more rapidly in early-maturing cultivars after flowering. Further, the maximum PSII photochemical efficiency and electron-transport rate in the silique wall are lower than those of late-maturing materials [41]. However, lower photosynthetic capacity at the single-silique level may not directly translate into yield limitation, because crop-level yield also depends on canopy light capture and planting density [42]. At an optimal density, the early-maturing cultivar Shengguang 127 showed 14.7–24.2% higher light-energy and temperature production efficiency than a medium-maturing control [43]. These results show that short growth duration can be compatible with high crop-level productivity under suitable planting density.

Harvest index (HI) is an important indicator for assimilate partitioning and the source–flow–sink balance, which directly reflects the proportion of above-ground biomass allocated to seed. A higher HI can partly compensate for lower total biomass [44,45]. In a rice–rice–rapeseed system in southern China, one early-maturing cultivar accumulated 11.2% less total dry matter than a conventional cultivar but had a 4.4% higher HI and an 8.2% higher seed yield [46]. Some extremely early spring-type materials have also shown higher HI and seed yield by enhancing the remobilization of reproductive organs [40]. Similar patterns have been observed for nutrient allocation. Some early-maturing materials show more efficient nitrogen remobilization from vegetative tissues to seeds. This is associated with higher proteolytic activity in vegetative organs, which maintains seed nitrogen supply despite lower total nitrogen uptake [47,48]. This sink-prioritized partitioning pattern helps maintain nitrogen supply to developing seeds, despite a shortened growth period.

### 2.2. Ecological Regulation

*Brassica napus* includes winter, semi-winter, and spring types [49,50]. Their differences in growth period are closely related to genetic background and environmental factors [18]. Temperature and photoperiod are the major environmental signals. Abiotic and biotic stresses may disrupt normal developmental progression [51]. Understanding these effects and their interactions is important for matching maturity to regional environments and cropping systems.

#### 2.2.1. Effects of Temperature and Photoperiod on Growth Period

Vernalization requirements differ among rapeseed ecotypes. Winter types have a strong requirement for prolonged low temperature, which relieves flowering repression mediated by *FLOWERING LOCUS C* (*FLC*) family genes. Semi-winter types generally have a shorter requirement that is sufficient for flowering, whereas spring types have little or no vernalization requirement [50]. Once the vernalization requirement is met, additional cold exposure does not necessarily accelerate flowering. Instead, it may coincide with declining freezing tolerance [38]. The extent of this decline differs among ecotypes. Winter rapeseed can retain some freezing tolerance, which gradually decreases during reproductive development [52]. In contrast, spring rapeseed loses freezing tolerance more rapidly after the reproductive transition, which partly explains its higher risk of spring cold injury under early-flowering conditions [53].

Unlike the typical response of high temperature promoting earlier flowering in *Arabidopsis*, the effect varies among ecotypes in *B. napus*. In spring rapeseed, most genotypes exhibit delayed flowering under sustained high temperature. *FLOWERING LOCUS T* (*BnaFTA2*) has been identified as an important gene in temperature response. The enrichment of the histone variant H2A.Z at the *BnaFTA2* promoter represses its expression, thereby delaying flowering under high temperature [54]. In semi-winter varieties, higher ambient temperature generally promotes flowering, whereas it may delay flowering in winter varieties [55]. These ecotype-dependent responses indicate that the flowering response to temperature in *B. napus* cannot be directly inferred from *Arabidopsis*.

Photoperiod is another important environmental signal regulating flowering. *B. napus* is a typical long-day crop. Long days promote flowering through upregulating the expression of *FT* family genes, whereas short days delay flowering [56]. Photoperiod sensitivity varies among ecotypes. Spring cultivars usually show stronger photoperiod responsiveness, while the response in winter varieties is relatively weaker due to the masking effect of vernalization requirements [57]. Within the same ecotype, photoperiod sensitivity shows continuous variation, providing useful genetic variation for growth-period improvement [57]. QTL mapping has identified loci affecting photoperiod responses. A locus on chromosome C01 advanced flowering by only 1.1–2.9 days under long days, but its advancing effect reaches 6.2 days under short days. The locus on chromosome C09 also is a specific locus, which exerted a flowering-promoting effect only under short-day conditions [58]. These loci provide key genetic resources for early-maturing breeding in low-latitude regions with short daylengths.

Temperature and photoperiod have significant interactive effects on flowering time. Under a 16 h photoperiod, 22 °C advanced flowering by 14 days relative to 11 °C. However, 22 °C delayed flowering by 15 days under an 8 h photoperiod. This result shows that the flowering response to temperature depends on day length. Genetic analysis also found that the temperature–photoperiod interaction explained more variation in flowering time than temperature alone. The loci on chromosomes A07 and C06 were identified as the core genetic loci mediating the temperature–photoperiod interaction [59]. Overall, early-maturing rapeseed breeding should consider suitable genotypes adapted to the temperature and photoperiod of the target planting region to achieve precise matching between growth period and the local environment.

#### 2.2.2. Relationships Between Stress and Maturity

Stress can affect the adaptation of rapeseed by changing flowering or maturity directly, or by altering the conditions experienced during sensitive developmental stages.

Drought can advance flowering in some species as a drought-escape response. In *Arabidopsis*, drought can activate *GIGANTEA* (*GI*), *FT*, *TWIN SISTER OF FT* (*TSF*), and *SUPPRESSOR OF OVEREXPRESSION OF CONSTANS1* (*SOC1*) through an ABA-dependent pathway, thereby promoting flowering and shortening growth period [60]. In *B. napus*, drought treatment advanced flowering from 77.00 to 75.05 days in a doubled-haploid population. QTLs for flowering time, root pull force, and yield co-localized on chromosomes A03, A10, and C02, indicating a genetic association among these traits under drought [61]. A QTL mapping study of drought at the bolting and budding stages identified 23 QTLs. Among them, a pleiotropic locus on chromosome A09 was associated with both flowering time and seed yield [62].

Unlike drought, salt stress generally delays flowering and maturity. In *Arabidopsis*, studies have revealed the GA-DELLA signaling pathway and the CO (CONSTANS)/FT photoperiod module involved in this process. Salt stress enhances DELLA-mediated growth inhibition and delays the transition to reproductive growth [63]. Meanwhile, salt stress downregulates the expression of *CO* and its downstream genes *FT*, *SOC1*, and *LEAFY* (*LFY*). Exogenous GA can partially alleviate the late-flowering phenotype caused by salt stress, suggesting that GA signaling is a key regulator of flowering time under salt stress [64]. In *B. napus*, high salinity under natural saline soil conditions delayed initial flowering by 4–5 days and maturity by 6–8 days. These changes were accompanied by the decline of leaf-area index, photosynthetic rate, and carbon–nitrogen balance. The inhibition of carbon assimilation and nutrient remobilization from vegetative organs to seeds are an important physiological basis for delayed developmental progression [65]. However, the molecular mechanisms remain unclear in rapeseed.

Waterlogging causes root hypoxia, a common stress for terrestrial plants. Deepwater rice can escape hypoxia through rapid internode elongation, restoring above-ground gas exchange [66]. However, current studies have mainly focused on the effects of waterlogging on growth and yield formation in rapeseed. Nabloussi et al. found that germination and seedling stages were most sensitive to waterlogging [67]. Wollmer et al. reported yield reductions after waterlogging at stem elongation and flowering, with small effects on seed quality [68]. The direct effects of waterlogging on growth period remain poorly understood in rapeseed.

Among biotic stresses, Sclerotinia stem rot (SSR) is a key constraint on stable production in rapeseed [69]. Ascospores of *S. sclerotiorum* usually infect senescent petals first. Infected petals then fall and remain on leaves, leaf axils, or stem surfaces, leading to stem lesions. High-humidity conditions elevate the risk of petal infection and disease development. Therefore, early-flowering rapeseed may be exposed to higher SSR risk, because its flowering period coincides with frequent rainfall and high humidity in early spring [70]. Genetic studies also report associations between SSR resistance and flowering time. In *B. napus*, QTLs for Sclerotinia resistance and flowering time were co-located on chromosomes A02, A03, C02, and C06 [71]. A negative correlation between flowering time and Sclerotinia resistance has also been reported, with earlier-flowering materials showing greater susceptibility. However, transcriptome comparisons of resistant and susceptible materials with similar flowering times indicate that resistance also has genetic determinants partly independent of flowering time [72]. CRISPR-mediated editing of *Inflorescence Deficient in Abscission* (*BnaIDA*) reduced silique shattering, floral organ abscission, and the spread of *S. sclerotiorum* [73]. This finding indicates that the floral organ abscission could be used as a target for improving SSR resistance in early-maturing rapeseed.

## 3. Genetic and Molecular Basis of Short Growth Duration in *B. napus*

**Figure 2:**
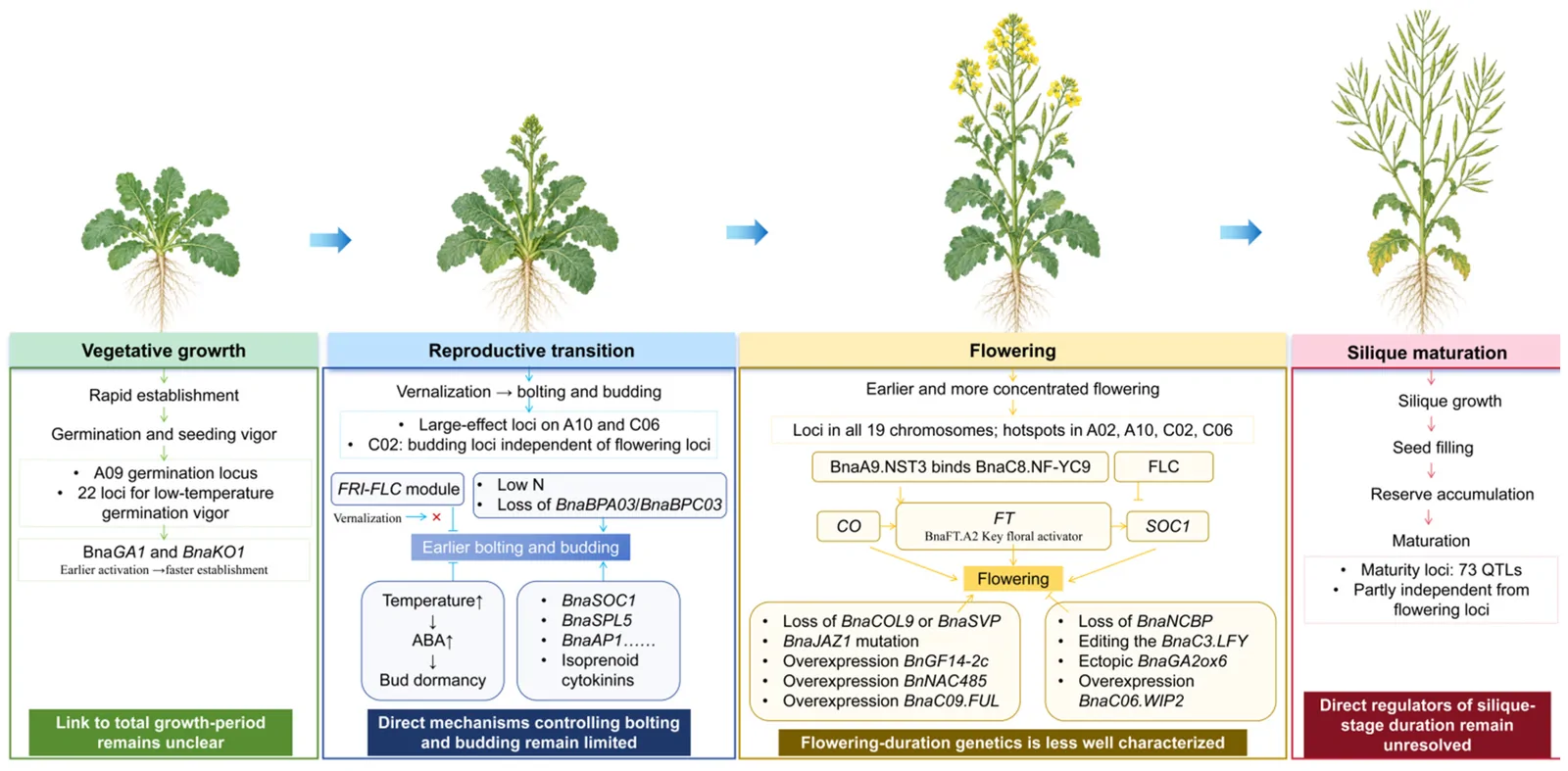
Stage-specific genetic and molecular basis of growth-period variation in *B. napus.* Growth duration consists of vegetative growth, reproductive transition, flowering, and silique maturation. This figure summarizes the representative loci and genes reported for each phase. The colored bar below each panel indicates the unresolved questions for that phase. ABA, abscisic acid; QTL, quantitative trait locus.

The growth duration of rapeseed is determined by multiple developmental phases. The short-duration period may be caused by faster-growing seedlings, earlier transition to reproductive development, more concentrated flowering period duration, or faster pod maturity period [17,74,75,76]. Although phase-specific traits are closely correlated, they also have partly independent genetic bases [17,18,75]. In recent years, genetic analyses and functional studies have identified loci and candidate mechanisms affecting several of these phases [20]. In *B. napus*, gene editing and mutagenesis have tested *BnaFT* paralogs, *BnaCOL9*, *BnaSVP*, *BnaNCBP*, *BnaC3.LFY*, and *BnTFL1* for effects on budding or flowering time [77,78,79,80,81,82,83,84]. Transgenic studies in rapeseed have also examined *BnaFLC.A10*, *BnaBPs*, *BnGF14-2c*, BnNAC485, *BnaC06.WIP2*, *BnaA9.NST3*, *BnPLP1*, *BnPtdIns-PLC2*, and *miR394* [85,86,87,88,89,90,91,92,93]. Near-isogenic lines and their hybrids have been used to assess combinations of *BnFLCs* alleles [24]. The flowering effects of *BnTOP1α-1*, *BnaGA2ox6*, and *BnC08.JAZ1-1* were tested in *Arabidopsis* [94,95,96]. Despite these advances, many genes proposed from QTL mapping and GWAS of growth-duration traits have not undergone comparable functional tests [17,18,74,97,98,99]. This section reviews the genetic loci and molecular regulatory mechanisms underlying four key developmental processes—vegetative growth, the vegetative-to-reproductive transition, flowering, and silique development and maturation (Figure 2).

### 3.1. Regulation of Vegetative-Phase Trait

For short-duration rapeseed, rapid and uniform germination and seedling emergence support timely establishment and early biomass accumulation. These traits are particularly important under late sowing, which further restricts the time available for crop growth. These traits are quantitative traits and generally controlled by many loci, most with small effects. A germination-related locus on chromosome A09 was linked to erucic-acid and glucosinolate content, implying the possibility that selection for double-low quality may have affected germination vigor [76]. Joint analysis identified co-localizing loci for seedling vigor and indole-3-acetic acid, ABA, and their metabolites [97]. Rapeseed lines with different germination speeds exhibited distinct dynamics of primary metabolites and hormone, such as malate, aspartate, ABA, GA, and IAA. In faster-germinating materials, *Gibberellin A1* (*BnaGA1*) and *KAURENE OXIDASE 1* (*BnaKO1*), were activated earlier [100]. Additionally, twenty-two loci associated with low-temperature germination vigor have been reported, with candidate genes related to DNA repair, mitochondrial activation, and storage-protein degradation [98]. However, whether these loci also affect the duration of later developmental phases remains unknown.

### 3.2. Regulation of the Vegetative-to-Reproductive Transition

Satisfaction of the low-temperature vernalization requirement is an important prerequisite for reproductive development in winter and semi-winter rapeseed. Current research about vernalization mainly focuses on the *FRI* (*FRIGIDA*)–*FLC* module in rapeseed. *FRI* acts as an upstream regulator of *FLC*. Its natural allelic variations are significantly associated with growth habit and flowering time, and the flowering time differs by 13–19 days among distinct haplotypes in rapeseed [101]. Structural variation and expression divergence among *FLC* paralogs contribute to variation in vernalization requirement. A Tourist-like miniature inverted-repeat transposable element (MITE) insertion in the upstream of the *BnFLC.A10* is strongly associated with a strong vernalization requirement [102]. Specifically, a 621-bp MITE element acts as a transcriptional enhancer in the promoter. The allelic combination with functional *BnaFLC.A2* contributes to the winter ecotype [85]. By contrast, a 2.833 kb insertion in the first intron of *BnFLC.A2* disrupts transcription and produces a loss-of-function allele. The loss-of-function alleles at both *BnFLC.A2* and *BnFLC.C2* significantly promotes early flowering [103].

After vernalization, the shoot apical meristem (SAM) acquires reproductive competence, followed by bolting and budding. Microscopic observation and transcriptome analyses of SAMs in winter rapeseed revealed that the transition occurred over several weeks after vernalization began. The process is accompanied by specific expression of *BnaSOC1*, *SQUAMOSA PROMOTER BINDING PROTEIN-LIKE 5* (*BnaSPL5*), and *APETALA1* (*BnaAP1*) homeologs [104]. Isoprenoid cytokinins also accumulate in the SAM during vernalization. They reach high levels when the transition becomes irreversible [39]. However, the transition does not necessarily result in immediate visible bud development. In winter rapeseed, the rising temperature can increase ABA content in flower buds and activate the *BRANCHED1*-associated dormancy module. This will delay budding and flowering in spring [55]. Nitrogen supply also modulates growth progression. The low-nitrogen treatment advances SAM differentiation by approximately 4–7 days relative to high nitrogen. The altered genes are involved in auxin biosynthesis, signaling, and floral meristem regulation during this process [35]. Functional analysis of the KNOX family genes *BREVIPEDICELLUS/KNAT1* (*BnaBPA03*) and (*BnaBPC03*) further links developmental timing with hormone regulation. Knockout plants showed earlier budding and flowering, increased GA and ethylene levels, and upregulation of hormone-biosynthesis genes and the floral integrators *BnaFT* and *BnaSOC1*, whereas overexpression delayed budding and flowering [86]. These findings connect the genetic regulation of reproductive transition with the hormonal processes discussed in Section 2.1.1, although the contribution of the accompanying hormone changes to the flowering phenotype remains to be resolved.

Research on the mechanisms of bolting and budding remains limited, and these traits are often analyzed together with flowering time. Multi-environment mapping in the Tapidor × Ningyou7 doubled-haploid population identified 16 QTL for bolting, 15 for budding, and 16 for initial flowering, seven of which affected all three traits. However, when budding and bolting time were included as covariates, most initial-flowering QTL effects were lost or substantially reduced. This indicates that some loci conventionally identified as flowering-time QTL actually exert their effects at earlier periods in reproductive development [105]. One region was subsequently fine-mapped to an approximately 80 kb interval on chromosome A10, and *BnFLC.A10* was identified as the major candidate gene [102]. Joint mapping of initial flowering, budding, and bolting identified 14 QTL, among which multiple loci on A10 and C06 exerted large phenotypic effects, and the C06 region contained overlapping QTL for all three traits [99]. A region on chromosome C02 was found to control both budding and initial flowering, but without overlapping. This implies the existence of independent regulators for budding [106]. GWAS in natural populations also identified stable loci, including *qFT.A07.1* (affecting both budding and bolting), *qFT.A10* (affecting bolting and initial flowering), and *qFT.C06* (specifically affecting budding), further supporting the genetic divergence among stages of reproductive transition [74].

### 3.3. Regulatory Network of Flowering Time

Flowering-time-related traits include the time to initial flowering and flowering duration. Initial flowering time is the most widely used indicator of evaluating growth period. It is a typical quantitative trait. Over one hundred flowering-time QTLs and QTNs have been identified across all 19 chromosomes, with the highest density on chromosomes A02, A10, C02, and C06. Some key regulating genes, such as *BnaA02.FT*, *BnaA10.FLC*, and *BnaA10.CO*, have been detected across multiple populations or environments and are potential targets for early-maturing breeding [20].

Linkage mapping, map-based cloning, and comparative genomics have characterized homeologs and allelic variation in core flowering-time regulators including *BnaCOs* [107,108,109], *BnaFLCs* [85,102,110], *BnaFRIs* [101], *BnaFTs* [77,111,112,113], *VERNALIZATION INSENSITIVE3* (*BnaVIN3*) [114], *BnaSOC1* [115]. With the development of transgenic technology and CRISPR/Cas9-mediated genome editing technology, the functions of some flowering genes have been characterized. While the effects of editing depend on the contributions of individual copies. For *SHORT VEGETATIVE PHASE* (*BnaSVP*), mutation of *BnaA09.SVP* alone did not change flowering time, whereas editing all four copies markedly advanced flowering [79]. Quadruple *CONSTANS-LIKE 9* (*BnaCOL9*) mutants also flowered earlier than wild-type plants [78]. Among *FT* paralogs, *BnaFT.A2* exhibited the strongest floral-promoting activity, followed by *BnaFT.C6* and *BnaFT.A7* [77], while a single mutation in *BnC6FTb* also can delay flowering, showing that editing several copies is not always required to alter flowering time [83]. Similarly, mutation of *BnaA03.NCBP* or *BnaA10.NCBP* alone delayed flowering, as well as double-copy *BnaNCBP* mutants [80]. Editing the second intron of *BnaC3.LFY* in the Hap2 background delayed flowering without editing other LFY homoeologs [81]. Thus, gene editing provides a way to test how individual homoeologs and their combinations affect flowering time. To identify combinations useful for breeding, the edited lines should also be evaluated for final maturity, yield, and other agronomic traits.

Transgenic overexpression and complementation have also been used to examine the effects on flowering time. Ectopic expression of *BnTOP1α-1* (*DNA topoisomerase Iα*) complemented the early flowering phenotype of *Arabidopsis top1α-7* mutant [94]. The overexpression of *BnGF14-2c* (*general regulatory factors 14-3-3s*) in the semi-winter rapeseed promoted flowering without vernalization [87]. Hormone-related regulators also affect flowering. The ectopic expression of *Gibberellin 2-oxidase* (*BnaGA2ox6*) in *Arabidopsis* delayed flowering by inactivating bioactive GAs [95]. Overexpression of *JASMONATE-ZIM DOMAIN 1* (*BnaJAZ1*) in *Arabidopsis* advanced flowering by approximately three days, indicating that jasmonate signaling may act as a potential floral repressor [96]. Overexpression of the ABA-induced transcription factor BnNAC485 accelerated flowering in *B. napus* and *Arabidopsis* and enhanced stress tolerance, suggesting a possible connection between flowering and abiotic-stress responses [88].

In recent years, with the deepening of research, the interactions between flowering genes have been reported. The floral activator *BnaC09.FUL* (*FRUITFULL*) directly interacted with the repressor *BnaC06.WIP2* (*WIP DOMAIN PROTEIN*), which represses the expression of downstream flowering genes [89]. BnaA9.NST3 (NAC SECONDARY WALL THICKENING PROMOTING FACTOR3) directly binds to the *BnaC8.NF-YC9* (*NUCLEAR FACTOR Y*) and *Bna.CO* promoters; moreover, BnaC8.NF-YC9 interacts with Bna.CO, forming NF-CO complexes that regulate *Bna.FT* transcription [90]. Despite this progress, the flowering regulatory network in *B. napus* remains far less resolved than in *Arabidopsis thaliana*.

Flowering duration also contributes to growth duration. In 300 rapeseed accessions, initial flowering time was strongly correlated with final flowering time (*r* = 0.705–0.777) but only moderately correlated with maturity (*r* = 0.512–0.570). Further GWAS analysis showed that these traits share partially overlapping loci, along with numerous trait-specific loci [17]. Wang et al. identified seven QTLs for flowering duration and 17 for initial flowering time, explaining approximately 7.95% and 17.48% of the phenotypic variation, respectively. Most growth-period QTLs were detected in only one year, suggesting substantial environmental sensitivity [18]. Haplotype analysis showed that *BnaELF4.A05-Hapla* was associated with a shorter flowering period, whereas *BnaLNK2.C06-Hapla* was associated with earlier final flowering and a shorter whole growth period. *BnaTOC1.C09-Haplc* was associated with earlier final flowering and a shorter flowering period, but a longer post-flowering maturation interval [18]. These associations suggest that different developmental phases can vary partly independently and that a shorter flowering period may be offset by a longer subsequent phase. Compared with initial flowering time, the genetic basis of flowering duration remains less well characterized.

### 3.4. Genetic Basis of Silique Development and Maturation

Post-flowering development includes silique growth, seed filling, reserve accumulation, and seed maturation. Seventy-three QTLs for maturity time have been identified, demonstrating the polygenic basis of maturity in rapeseed [22]. Although initial flowering time and maturity are significantly correlated, they also have trait-specific loci [17]. Mechanistic studies of post-flowering development have focused mainly on assimilate supply and reserve accumulation rather than on the duration of maturation [116,117,118,119]. Genes directly controlling silique-period duration or physiological maturity remain poorly characterized.

From a practical breeding perspective, loci that only regulate flowering time may produce early-flowering phenotype. However, this advantage may not translate into earlier maturity if it prolongs later reproductive phases. Therefore, genetic variants that shorten multiple development phases without substantial yield penalties may have greater value for breeding rapeseed with a short growth duration.

## 4. Genetic Relationships Between Growth Duration and Other Target Traits in Rapeseed

### 4.1. Genomic Co-Localized Loci

**Figure 3:**
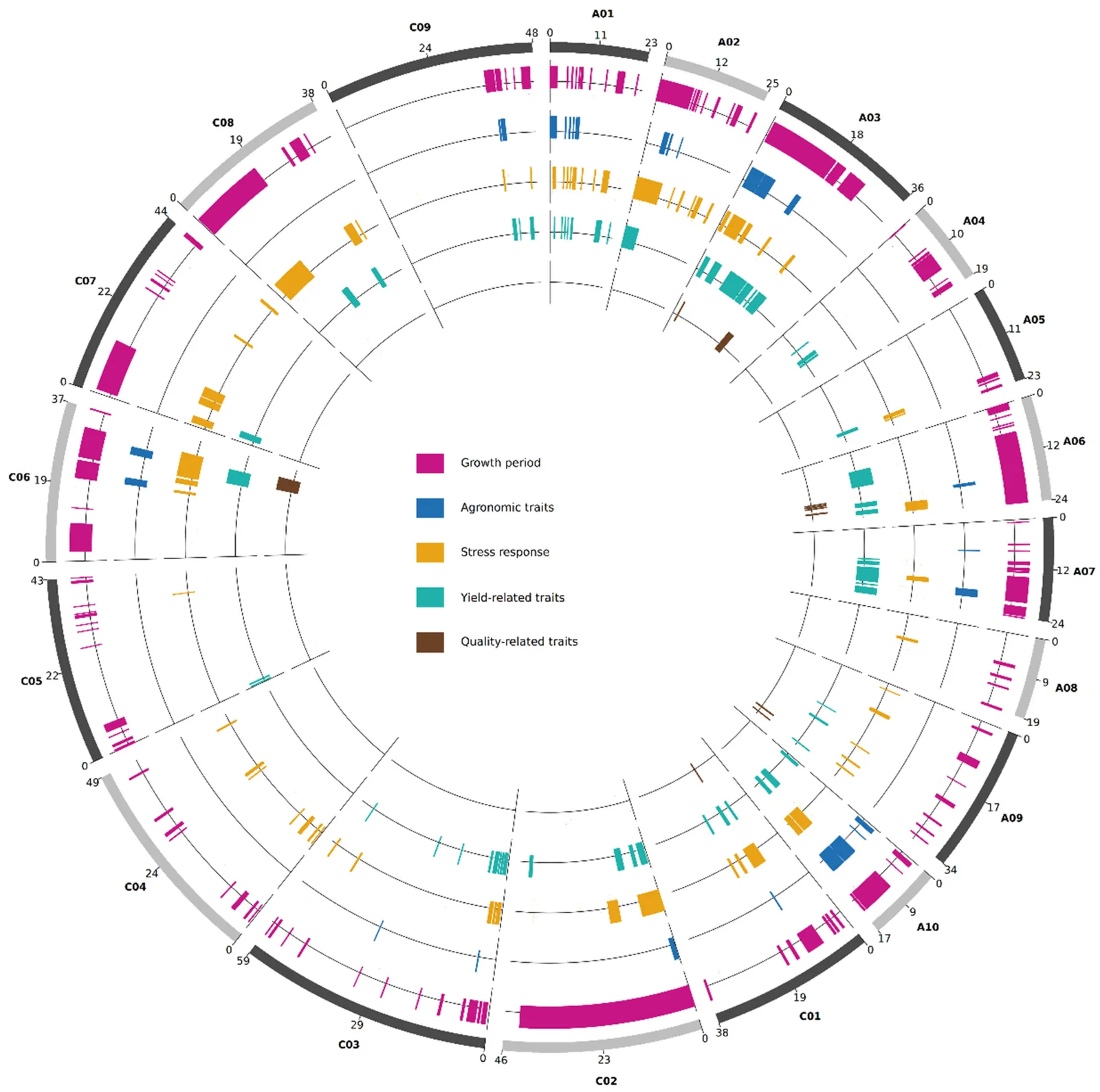
Genome-wide overlap of reported growth-duration loci with loci for other traits in *B. napus*. The outermost track represents the 19 chromosomes of the *B. napus* genome, with physical coordinates in megabases (Mb). The five concentric inner tracks show the physical intervals of reported loci associated with growth duration (magenta), agronomic traits (blue), stress responses (orange), yield-related traits (teal), and quality-related traits (brown), from the outermost to the innermost track. Overlapping intervals across tracks indicate genomic co-localization.

Growth-duration related traits are quantitative and may affect seed yield and quality directly or indirectly [74]. Genetic loci for yield-related traits [22,120,121], quality-related traits [122], and flowering time [123,124] have been reviewed. However, the overlap with growth duration-related traits remains poorly characterized. Here, we compiled published genetic loci in rapeseed. These loci were classified into five categories, including growth duration, yield, quality, stress response, and agronomic traits (Table S1) [17,18,22,23,49,50,58,59,71,74,99,106,119,125,126,127,128,129,130,131,132,133,134,135,136,137,138,139,140,141,142,143,144,145,146,147,148,149,150,151,152,153,154,155,156,157,158,159,160,161,162,163,164,165,166,167,168]. All loci were aligned to the physical map of “Darmor-*bzh*” reference genome [169]. Co-localization analysis was then performed to reveal genetic associations between growth duration and other traits (Figure 3).

Growth-duration loci distributes across all 19 chromosomes, with relatively high densities on A02, A03, A10, C02, and C09 (Figure 3 and Table S1). These regions are not fully consistent across studies. For instance, major growth-duration QTLs on C02 and C06 remained stable across multiple environments. In contrast, the A03 region is mainly detected in semi-winter ecotypes and a C08 region across different environments [125,129]. Among the published records in each trait category, 144 of 229 yield-related records (62.9%), 96 of 275 seed-quality intervals (34.9%), 1913 of 5714 stress-response intervals (33.5%), and 56 of 389 agronomic-trait intervals (14.4%) overlap at least one growth-duration interval (Table S1). Growth-duration loci extensively overlap with reported quality- and stress-response loci on nearly all chromosomes. Overlaps with agronomic traits are concentrated on chromosomes A01, A03, and A10, whereas overlapping with yield-related traits are distributed on all chromosomes except A08 and C04. The A subgenome contains more reported overlaps than the C subgenome.

Physical overlap between loci can arise through pleiotropy, linkage, or indirect developmental effects. For example, a doubled-haploid population showed co-localized QTLs for flowering time, plant height, branch initiation height, and stem diameter on A02 and A07. Conditional QTL analysis suggested both pleiotropy and linkage [170]. Several flowering regulatory genes have been found to affect other traits simultaneously [24,86,91]. Further, co-localization can also arise from developmental cascade effects between traits. A systematic analysis of 21 traits identified 134 consensus QTLs. Conditional QTL mapping confirmed that the hub-QTL cluster QC4 on chromosome A02 had the most direct effect on growth duration. After controlling for growth duration as a covariate, its effects on plant height, primary branch number, silique number, seeds per silique, and seed weight declined markedly. This indicates that the impact of QC4 on yield is mediated by changes in growth duration, which cascades into downstream effects on vegetative growth, plant architecture, and reproductive development [171]. In natural populations, markers near the flowering regulator *FT* are associated with plant emergence, shoot biomass, and seed yield. The effects of the *FT* haplotype on these traits vary across environments [172].

Genetic intersections between growth duration and stress responses may reflect shared loci, linkage, or alignment of sensitive developmental stages with stress exposure. Among biotic stresses, Sclerotinia stem rot is one of the most devastating diseases in rapeseed. Multiple studies have found significant co-localization between Sclerotinia stem and flowering time. Thirty Sclerotinia-resistance and 22 flowering-time QTLs simultaneously mapped in a RIL population [173]. Similarly, co-localization clusters for the two traits were detected on chromosome A02 and C02 [135]. However, not all Sclerotinia resistance are affected by flowering time. Conditional analyses in some doubled-haploid populations found resistance QTLs with no significant dependence on flowering time [174]. Therefore, Sclerotinia resistance and flowering time may involve shared and partly independent genetic control.

In terms of abiotic stress, there are distinct adaptation strategies. For example drought stress, earlier flowering may reduce exposure through drought escape, whereas later flowering and a larger root system may contribute to dehydration avoidance [175]. Joint analyses have identified regions associated with root traits, flowering time, and yield under irrigated and rainfed conditions. Conditional models further distinguish the direct effects on root traits from their indirect effects through flowering time [61]. Under terminal water-deficit conditions, flowering-time and yield QTLs co-localized on A09. This region also overlaps with QTLs for water use efficiency [62]. GWAS of salt tolerance at the germination and seedling stages have also identified intervals overlapping with growth-period loci [144].

Taken together, growth period shows extensive genomic co-localization with yield, quality, agronomic architecture, and stress response in rapeseed. Such overlap may reflect pleiotropy, linkage, or developmental mediation. Pleiotropy may constrain independent improvement, whereas recombination may separate linked loci [171,176]. Importantly, physical co-localization alone only demonstrates genomic proximity and cannot distinguish among these mechanisms. Fine mapping, conditional analyses, near-isogenic materials, and functional validation are needed to resolve causal relationships.

### 4.2. Candidate Genes Associated with Growth Duration and Other Traits

**Figure 4:**
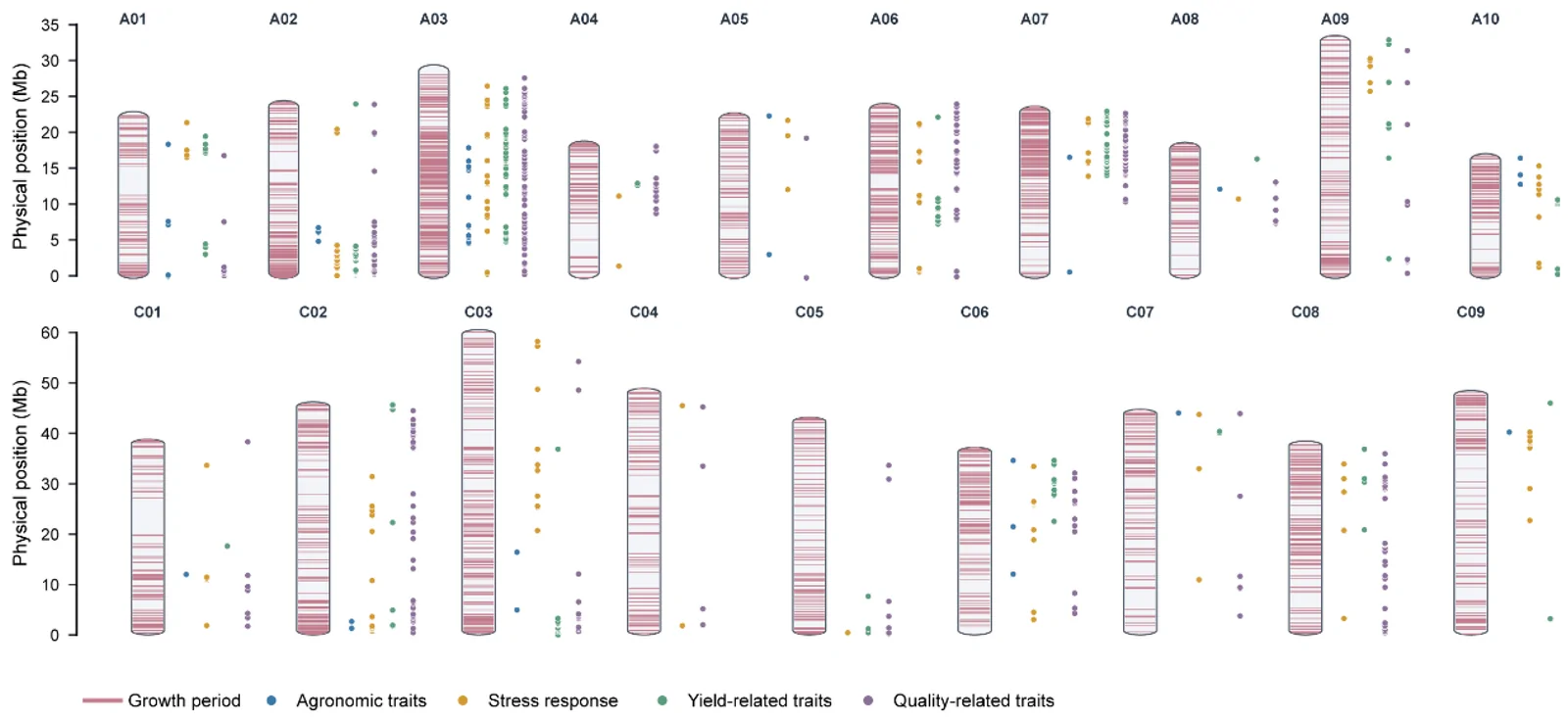
Chromosomal distribution of candidate genes between growth period-related and other traits in *B. napus.* Genes are mapped to the 19 chromosomes of *B. napus* by physical position (Mb). Red lines represent genes associated with growth period. Colored dots indicate genes that simultaneously associated with other traits.

Among the 2923 growth-duration-related candidate genes, 904 (30.9%) of which also appear among candidates for other traits (Figure 4 and Table S2), the highest proportions are found on chromosome A03, accounting for 61.5%, followed by A02 (60.3%) and A07 (55.4%) (Table S2) [20,22,36,84,86,87,91,119,139,140,141,143,144,145,146,147,148,149,151,152,153,154,156,157,158,159,160,161,162,163,164,165,167,171,177,178,179,180,181,182,183,184,185,186,187,188,189,190,191,192,193,194,195,196,197,198,199,200,201,202,203,204,205,206,207,208,209,210,211,212,213,214,215,216,217,218,219,220,221,222,223,224,225,226,227,228,229,230,231,232,233,234,235,236,237,238,239,240,241,242,243,244,245,246,247,248].

#### 4.2.1. Pleiotropy of Flowering Regulatory Genes

Flowering-time regulatory genes are the most extensively studied associated with growth duration. Functional studies in several crops show that flowering regulators can also affect plant architecture, yield components, and quality.

In rice, heading-date genes can also affect yield components and plant architecture. *Grains.Height.Date-7* (*Ghd7*) gene delays heading under long days and affects plant height and panicle traits [249]. *Nuclear transcription factor Y subunit B* (*DTH8*) similarly suppresses flowering while influencing plant height and yield potential. It delays flowering under long days by regulating *Early heading date1* (*Ehd1*) and *Heading date-3a* (*Hd3a*), and increases tiller and branch number by upregulating *MONOCULM 1* (*MOC1*), thereby enhancing grain number per plant [250,251]. *Ghd7.1* also significantly delays heading and increases yield under long-day conditions [252]. CRISPR analyses of heading-date genes have reported distinct effects on yield components depending on the allele combination and genetic background [253,254]. In wheat, natural allelic variation at *Vernalization 1* (*Vrn-1*) and *Photoperiod 1* (*Ppd-1*) affects heading date and agronomic traits, with allelic combinations producing different trade-off patterns between earliness and yield potential [255,256]. In soybean, different allelic combinations of *E1*–*E4* simultaneously affect flowering time, maturity, plant height, internode length, yield per plant, and seed quality. *E1* also participates in a pathway linking flowering and stem termination [257,258]. In *Brassica juncea*, reduced expression of *SOC1* delayed flowering and altered branching, yield components, and fatty-acid composition [259].

In *B. napus*, CRISPR/Cas9 knockout of *BnaC03.TFL1* caused early flowering and reduced plant height, branch-initiation height, branch number, silique number, and seed number per silique [82]. An EMS-induced *BnTFL1-2* mutant had limited effects on flowering time but altered seed number per silique and total seed number. Its F_1_ hybrid derived with the non-mutated parent showed improved yield components [83]. Systematic knockout of six *BnTFL1* paralogs caused early flowering and different plant architecture, whereas seed yield is not significantly affected in single, double, or triple mutants. The result indicates that yield loss associated with early flowering can be decoupled from editing specific TFL1 copy combinations [84]. Overexpression of *BnPLP1* causes earlier flowering initiation, prolonged flowering duration, increased plant height, and longer main inflorescence in *Arabidopsis*. However, the downregulation of *BnPLP1* in rapeseed produces opposite phenotypes [91]. A GWAS study identified *BnFT* on A02 as a candidate gene for plant-height regulation [155]. In *B. juncea*, natural variation in *FT* homeologs also was associated with silique shape, oil profile, stomatal morphology, and plant height [260].

Specific alleles and their combinations may help achieve earlier development while maintaining or improving yield. *BnFLC.A2*, *BnFLC.C2*, and *BnFLC.A3b* had been identified the association with flowering-time variation and yield per plant. Some near-isogenic line hybrids carrying appropriate allelic combinations earlier flowering while maintaining or increasing yield [24]. Yin et al. introgressed the reduced-function *BnaFLC.A2^Wes^* haplotype into a semi-winter background. The resulting near-isogenic lines flowered approximately 20 days earlier in Wuhan field trials and maintained relatively stable seed yield and oil content under normal sowing and a 17-day delay in sowing [261]. Wang et al. combined the early-flowering loci *qFTA10* and *BnaC08cqDTF* through marker-assisted selection to develop line 195 from N78 × N133, with *BnCRY2* identified as the candidate gene underlying *BnaC08cqDTF.* Across five high-altitude sites over two consecutive years, the mean yield of line 195 exceeded that of the locally cultivated *B. rapa* cultivar Haoyou 11 by 23.07% [262].

#### 4.2.2. Collateral Effects of Other Trait Genes on Growth Duration

Genes primarily studied for stress response, yield, or quality can also affect growth duration. Overexpression of *Phosphatidylinositol-specific phospholipase C 2* (*BnPtdIns-PLC2*) in rapeseed enhanced drought tolerance, promoted the reproductive transition, and shortened time to maturity, alongside changes in photosynthesis, stomatal behavior, and hormone distribution [92]. The expression of NAC transcription factor *BnNAC485* is induced by drought, high salinity, and ABA. Its overexpression enhances abiotic stress tolerance in both rapeseed and *Arabidopsis* and causes earlier flowering and alters expression patterns of several circadian clock genes [88]. Overexpression of *miR394* enlarged vegetative and reproductive organs, as well as altering seed quality traits and delaying flowering time [93].

Overall, research on the coordinated regulation of growth duration and other traits remains limited in *B. napus*. Most studies have been restricted to QTL/QTN localization or focus on individual traits. Therefore, it is critical to mine favorable allelic variants of these pleiotropic loci and explore the coordinated mechanism of growth duration and target breeding traits in rapeseed.

### 4.3. Phenotypic Performance of Early-Maturing Strains in the 2021–2025 Chinese National Regional Trials

To characterize the phenotypic performance of current early-maturing breeding materials, we analyzed records for 99 breeding lines entered in the early-maturity group of the Chinese National Winter Rapeseed Regional Trials conducted from 2021 to 2025 [25,26,27,28,29]. Twelve entries were evaluated in each of 2021 and 2022, and the number of entries increased from 2023 onward (Table S3). This reflects the increasing attention for short-duration cultivars.

#### 4.3.1. Phenotypic Variation and Relative Performance Across Years

**Figure 5:**
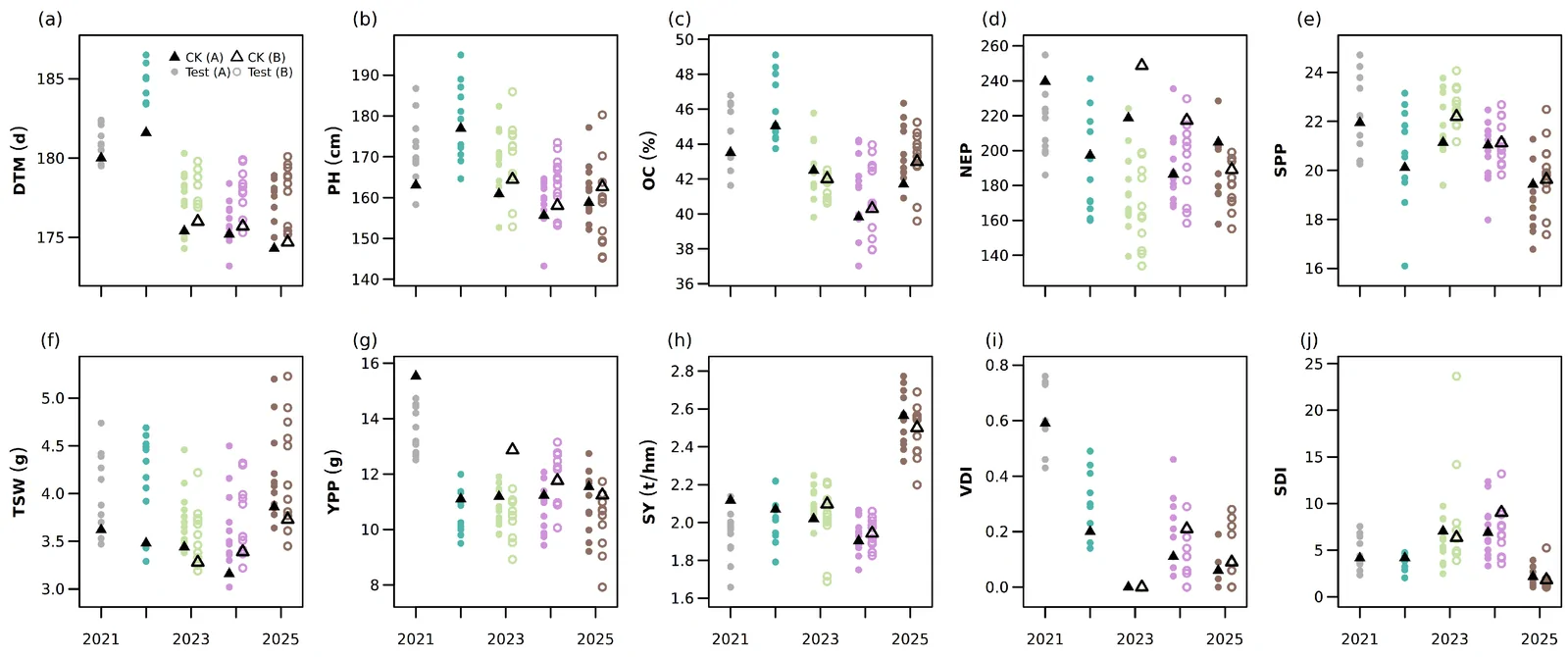
Phenotypic variation in national early-maturity rapeseed trials. Each point represents the mean trait value of a line across 8 or 9 trial locations in the corresponding year. Only group A was evaluated in 2021 and 2022, group B was added from 2023 onward, and each group had its own control. Circles represent test lines and triangles represent controls. Filled and open symbols denote groups A and B, respectively. Panels show (**a**) days to maturity (DTM, d), (**b**) plant height (PH, cm), (**c**) oil content (OC, %), (**d**) effective siliques per plant (NEP), (**e**) seeds per silique (SPP), (**f**) thousand-seed weight (TSW, g), (**g**) yield per plant (YPP, g), (**h**) seed yield (SY, t/ha), (**i**) virus disease index (VDI), and (**j**) Sclerotinia disease index (SDI). Data were compiled from the annual trial reports for 2021–2025.

Among the measured traits, days to maturity (DTM), thousand-seed weight (TSW), and oil content (OC) showed relatively consistent patterns relative to annual controls. Within-year DTM coefficients of variation ranged from 0.58% to 0.94%. Mean DTM of test stains was 0.78–3.24 days longer than that of the corresponding controls, and only approximately 11% of the lines matured earlier (Figure 5a and Table S3). These results suggest that most entries were not earlier-maturing than the controls, while selection appears to have maintained or improved other traits within the tested maturity range. TSW was the most consistent advantage of the test lines relative to the controls. Across the five years, 77.3–87.5% of the lines exceeded their corresponding controls by 0.29–0.70 g, and the maximum TSW increased from 4.74 g in 2021 to 5.23 g in 2025 (Figure 5f and Table S3). In a separate field study, early-maturing cultivars matured 3–5 days earlier than medium-maturing controls and maintained high seed yield under optimal planting density. This performance was associated with greater seed weight and higher light- and temperature-use efficiencies [43]. The annual mean OC ranged from 41.02% to 45.98%, with a CV of 3.20–5.27%. In four of the five seasons, the mean OC of the test lines exceeded that of the corresponding controls by 0.93–0.97 percentage points. The highest OC observed among the test lines was 49.10% (Figure 5c and Table S3). These results demonstrate that some short-duration materials can maintain high oil content.

Annual changes in plant height (PH) largely paralleled with the corresponding controls. Mean PH of the test lines was higher than that of the control in most groups. However, the proportion of lines with a plant height of ≤160 cm increased in the later trials (Figure 5b and Table S3), indicating that the shorter materials became more frequent within the early-maturing breeding. The Sclerotinia disease index (SDI) varied substantially among years and entries. This variation may reflect differences in rainfall during flowering, petal and leaf infection, and canopy microclimate [70].

For yield components, mean TSW of the test lines exceeded that of the controls, whereas the mean pod number per plant (NEP) and yield per plant (YPP) were lower than the controls for most stains (Figure 5d–h and Table S3). Thus, the increase in seed weight did not fully offset the lower silique number per plant in these comparisons. Rapeseed yield is determined by silique number, seeds per silique, and seed weight, with compensatory relationships among components [138,263]. A previous review reported a seed-yield range of 1.64–2.22 t ha^−1^ for short-duration winter rapeseed [8]. By comparison, the annual mean seed yield ranged from 1.93 to 2.77 t ha^−1^ during 2021–2025, with most falling within the previously reported range. The proportions of lines equaling or exceeding the yield of the corresponding control were 9.1%, 36.4%, 56.5%, 54.2%, and 50.0% from 2021 to 2025, respectively (Figure 5h and Table S3). Thus, the proportion reaching control-level yield was higher from 2023 onward than in the first two years. Due to the increase in the oil content of the test lines, the increase in oil production per unit area is greater compared to the control group. In total, 75.0%, 75.0%, and 72.7% of the group entries equaled or exceeded the control in 2023, 2024, and 2025, respectively. Mean oil yield in these annual comparisons was 3.1–5.2% higher than that of the corresponding controls (Table S3).

Overall, the current improvement of early-maturing rapeseed is increasingly focused on multiple traits within an established maturity window. Larger seed weight and higher oil content were common relative advantages, but population-level yield also depended on silique number, seeds per silique, and stand performance. The identification of the stable resistance to Sclerotinia across environments should also be integrated into this multi-trait selection framework.

#### 4.3.2. Trait Correlations and Yield-Component Relationships

**Figure 6:**
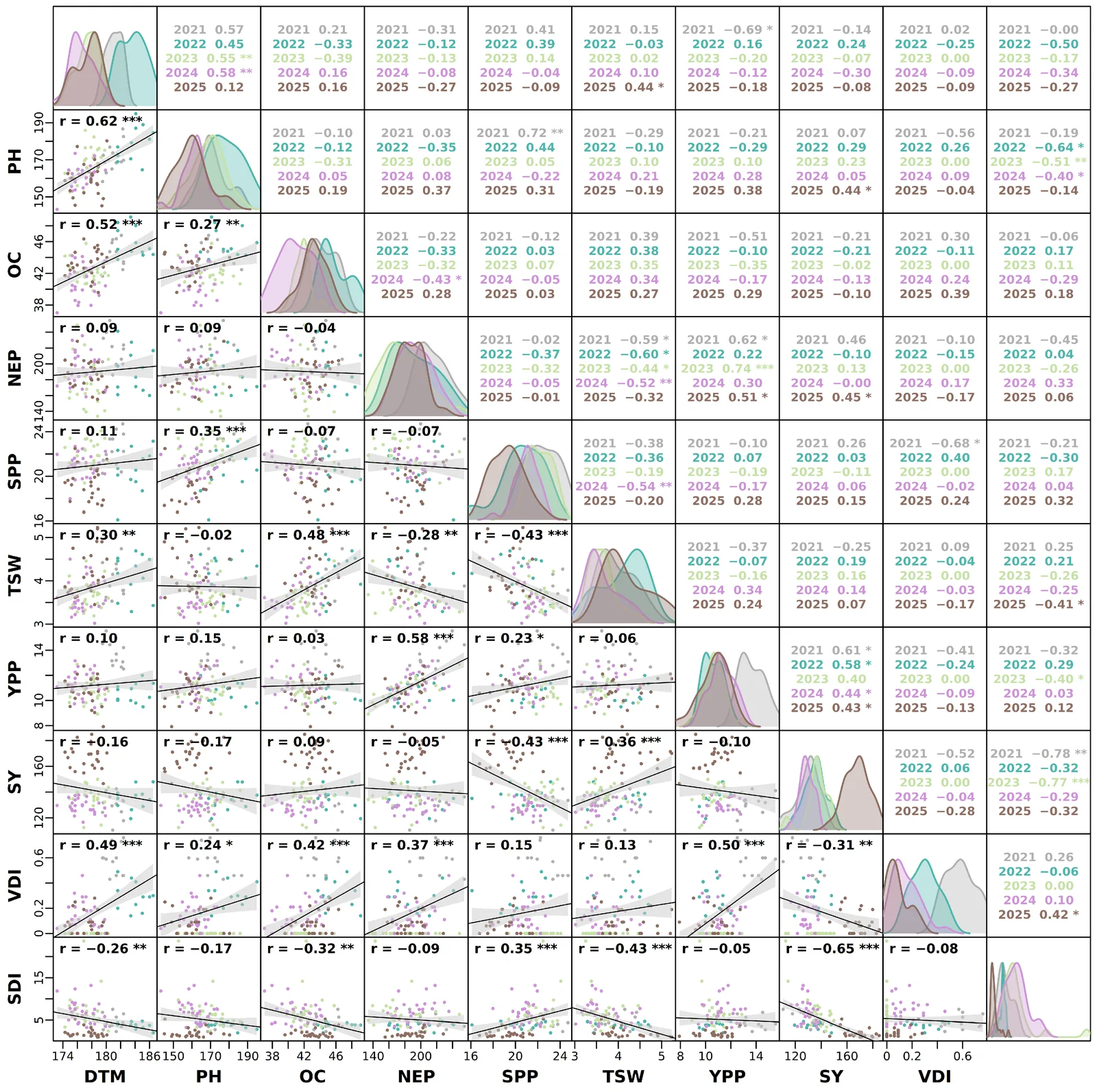
Correlations among traits in national early-maturity rapeseed trials. Diagonal panels show kernel-density distributions for each trait by harvest year. Colors distinguish harvest years. The lower triangle shows pooled scatterplots and Pearson correlation coefficients (*r*) based on the 2021–2025 trial data, including test lines and their group-specific controls. Each point represents the mean for one entry across 8 or 9 trial locations. Solid lines indicate linear regression fits, and gray shading indicates 95% confidence intervals. The upper triangle reports Pearson correlation coefficients (*r*) calculated separately for each year. Traits are days to maturity (DTM, d), plant height (PH, cm), oil content (OC, %), effective siliques per plant (NEP), seeds per silique (SPP), thousand-seed weight (TSW, g), yield per plant (YPP, g), seed yield (SY, t/ha), virus disease index (VDI), and Sclerotinia disease index (SDI). Asterisks indicate significance levels, * *p* < 0.05, ** *p* < 0.01, and *** *p* < 0.001.

Pearson correlation analysis showed that DTM and PH had the most consistent association among relationships involving DTM. The pooled correlation coefficient across five years was 0.62 (*p* < 0.001). The annual coefficients ranged from 0.12 to 0.58 and were significance in 2023 and 2024 (Figure 6). Longer duration tended to accompany higher plant height in the pooled data, although the relationship varied among years (Figure 6). Previous studies also reported positive correlations and overlapping QTLs for flowering time and plant height in rapeseed [170,264]. However, PH was not significantly correlated with SY across years in these groups, implying that plant height and yield may be amenable to independent improvement.

Annual correlation coefficients between DTM and SY ranged from −0.30 to 0.24 and were not statistically significant (Figure 6), demonstrating that extending growth duration in the early-maturity group did not lead to increased seed yield per unit area. The relationship between DTM and OC also varied among years, with no consistent direction. In contrast, the association between DTM and SDI was weakly negative in all years (Figure 6), suggesting that earlier-maturing materials tended to be slightly more susceptible to Sclerotinia. Previous studies have shown that early-maturing materials flower relatively early. Sclerotinia infection typically occurs two to three weeks after flowering under warm, rainy conditions, petal-borne inoculum. Therefore, early flowering may contribute to the slightly heavier disease [70,135].

Among yield components, NEP and SPP were both negatively correlated with TSW, whereas NEP was positively correlated with YPP, and YPP was positively correlated with SY in all years (Figure 6). In contrast, annual correlations between TSW and SY were weak and not significant (Figure 6). Although higher TSW was the most consistent advantage of the test lines over the corresponding controls, this advantage alone did not ensure higher population-level yield. This is consistent with the established contribution of silique number, seeds per silique, and seed weight to rapeseed yield [138,263,265]. Notably, SDI was negatively correlated with SY in all five years (*r* = −0.78 to−0.29), with significant associations in 2021 and 2023 (Figure 6). Field trials have documented yield losses associated with Sclerotinia infection [266]. Therefore, further yield improvement within this early-maturity germplasm will require evaluation of seed weight and silique number, coupled with the introgression of Sclerotinia resistance that does not delay maturity.

To further identify the relationships of these traits with YPP, SY, and OC, we performed path analysis with standardized coefficients (Table 1). The result shows that NEP, SPP, and TSW had positive direct paths to YPP. All three had positive indirect effects on SY through YPP, but no significant direct effects on SY. This indicates that NEP, TSW, and SPP influence SY mainly by increasing YPP. NEP and SPP were negatively correlated with TSW in Figure 6, and the annual TSW–SY correlations were weak. This helps explain why the test lines’ higher TSW did not ensure higher SY by itself. The pattern reflects compensation among yield components [138,263,265]. TSW also had a positive direct path to OC (Table 1), consistent with the generally higher OC observed in the test lines than in their corresponding controls (Figure 5).

SDI showed the strongest direct negative effect on SY but no significant effect on YPP (Table 1). This indicates that disease reduces SY through stand-level effects rather than by lowering individual plant performance. It is consistent with the consistently negative SDI–SY correlations across years (Figure 6) and reported yield losses from Sclerotinia infection [266]. DTM had a negative direct effect on YPP and a corresponding negative indirect effect on SY through YPP, but its direct effect on SY was not significant. The annual DTM–SY correlations varied in direction (Figure 6). After controlling for these factors, longer DTM within the early-maturity range showed no direct advantage for SY. Overall, maintaining plant-level yield and limiting Sclerotinia pressure are priorities for increasing SY in these early-maturity lines. Neither higher TSW alone nor a longer maturity period showed a clear area-yield advantage.

## 5. Conclusions and Perspectives

Short-growth-duration rapeseed can extend the use of winter fallow land, thus contributing to domestic edible-oil supply. In rice–rapeseed rotations in the Yangtze River basin, short-growth-duration cultivars can help fit rapeseed into the interval before the following crop. In spring-rapeseed regions, it also supports adaptation to a short growing season. Previous studies of growth-duration traits have largely focused on flowering time, and their regulatory networks have been extensively characterized [20,123,124]. However, initial flowering time is only moderately correlated with maturity. The genetic loci also overlap only partly, and some loci show contrasting effects at different developmental stages [17,18], implying this indicator does not fully represent maturity. Earliness should therefore be defined as an adaptive phenotype expressed across the whole growth period. The key indicators should include bolting and budding time, initial and final flowering time, seed-filling duration, and days to physiological maturity. Further, evaluation should combine with the temperature, light conditions and cropping system of the target production region.

Previous studies have provided initial insights into the basis of short growth duration and its coordination with other traits. Physiologically, a lower C/N threshold for floral bud differentiation, a higher GA/ABA ratio, and stronger post-flowering remobilization of assimilates to seeds help early-maturing materials maintain a higher harvest index and sustain yield formation within a shorter growth period [32,33,46]. Ecologically, temperature and photoperiod responses differ among ecotypes and interact to affect flowering [50,59]. Drought can advance flowering as an escape response, whereas salinity delays the reproductive transition [51]. Early flowering under humid spring conditions will raise the risk of Sclerotinia infection [70]. Genetically, most advances concern the *FRI*–*FLC* vernalization module and the *FT*-mediated flowering network [77,102,103]. These studies about *TFL1* and *FLC* alleles also indicate that maturity can be modified without an inevitable reduction in yield potential [24,84]. Hormone regulation provides one connection between the physiological, ecological, and genetic findings reviewed here. Winter warming increased flower-bud ABA levels and delayed flowering in winter rapeseed [55], whereas disruption of *BnaBPs* altered hormone levels and flowering-related gene expression alongside earlier reproductive development [86]. At the mapping level, joint analysis of germination, seedling vigor, and hormone contents identified QTLs for developmental and physiological traits within the same population [97]. Beyond flowering, *BnPtdIns-PLC2* overexpression was accompanied by altered hormone distribution and photosynthetic characteristics, together with earlier maturation [92]. These studies connect particular physiological processes with environmental responses or genetic variation. However, the extent to which growth-duration loci act through changes in the GA/ABA balance or carbon–nitrogen status around floral transition remains insufficiently resolved.

Data from the early-maturity group of the 2021–2025 Chinese national regional trials show that larger seed weight and higher oil content were common advantages over the established high-yielding controls (Figure 5). However, nearly half of the test lines did not reach the control yield, mainly because of fewer effective siliques per plant and fewer seeds per silique (Figure 5 and Table S3). The negative correlation between NEP and TSW reflects compensation among yield components [138,263]. Although the test lines had larger seeds, this advantage was offset by fewer siliques per plant. Path analysis confirmed that NEP and TSW influenced SY mainly through their positive effects on YPP. SDI had the strongest direct negative effect on SY (Table 1). Within the tested maturity range, longer DTM showed no direct advantage for SY. TSW also had a positive direct effect on OC (Figure 6 and Table 1), and in some trial groups higher OC partly offset lower SY in terms of oil yield per unit area. Further improvement should therefore focus on increasing silique number and reducing Sclerotinia pressure while maintaining seed weight, oil content, and the target growth duration.

Future work should resolve the basis of whole-growth-period traits in different production ecologies. The central question is not merely which genes advance flowering, but which determine final maturity and whether they can be modified without impairing yield, quality, and resistance. Therefore, the phenotypic analysis should quantify the timing of reproductive transition, the duration of flowering, and the seed-filling duration. Simultaneously, the yield-, quality-, and resistance-related traits should also be evaluated under the same genetic background and experimental conditions. Physiological traits should also be measured in these populations, including GA and ABA contents and their ratio, carbohydrate and nitrogen status around floral transition, and assimilate remobilization during seed filling. Measurements at both matched chronological ages and comparable developmental stages would help distinguish physiological changes preceding a developmental transition from those resulting from it. Joint mapping could identify regions associated with both physiological traits and developmental timing. Subsequent analyses in near-isogenic or edited materials, combined with hormone or nutrient treatments, could test whether the physiological changes mediate the developmental effects of particular alleles.

The association between these maturity components and other traits may arise from pleiotropy, genetic linkage, or indirect developmental effects. Joint mapping can identify shared regions. Conditional analysis can test whether associations persist after accounting for a maturity component. Fine mapping, near-isogenic lines, and functional validation are also required for causal resolution. Unfavorable linkage may be broken by selecting recombinants that retain favorable alleles. When one gene affects several traits, natural alleles or edited homoeolog combinations can be compared for their effects on maturity and other breeding traits. After favorable allele effects have been confirmed in breeding materials, allele-specific markers can support their transfer and combination. For traits controlled by many loci, genomic selection may help rank lines jointly for maturity and agronomic performance. Promising lines should then be evaluated in target production environments.

Resolving these loci in the allotetraploid genome of *B. napus* requires approaches that account for functional redundancy among homeologs and gene dosage effects. Gene editing enables functional comparisons of single- and multiple-copy mutants, as the studies of *BnaSVP*, *BnaNCBP*, and *BnTFL1* [79,80,84]. Functional studies of *FLC* and *TFL1* provide a reference for evaluating homeolog-specific and dosage effects in *B. napus* [24,84]. Pangenome and structural-variation analyses can identify haplotypes across ecotypes [50,114]. Transposon insertions in *BnaFLCs* and *BnaFT.A2* are associated with flowering time and seasonal crop type [85,113]. Insertion-specific markers could be developed to track these alleles during breeding and help select lines for evaluation in the target growing region. Additionally, excellent alleles and their combinations should be evaluated across multiple production environments. This result will determine whether the allele has broad breeding value or should be deployed only in a defined production ecology. Early-maturity breeding should prioritize stage-specific effects and environmental stability, rather than relying only on average effects on flowering or maturity. The ultimate objective is to obtain genotypes that reach the required maturity window while maintaining effective silique formation, seed filling, oil content, and resistance to Sclerotinia stem rot.

## Figures and Tables

**Table 1:** Standardized direct and indirect path coefficients for YPP, SY, and OC.

Trait	YPP	SY Direct	SY via YPP	OC
DTM	−0.310 *	−0.148	−0.102 *	−0.111
NEP	0.700 *	−0.009	0.230 *	0.078
PH	0.128	0.081	0.042	0.002
SDI	−0.053	−0.433 *	−0.017	−0.026
SPP	0.311 *	0.128	0.102 *	0.206
TSW	0.558 *	0.040	0.183 *	0.466 *
VDI	−0.103	−0.084	−0.034	0.206
YPP	—	0.328 *	—	−0.207
R^2^	0.437	0.361	—	0.127

Note: Year-by-group effects were included, and coefficients were standardized within groups. Indirect paths to SY were calculated as the product of the path from each trait to YPP and the path from YPP to SY. Confidence intervals are percentile intervals from 3000 bootstrap resamples within year-by-group sets. * *p* < 0.05. DTM, days to maturity; NEP, effective siliques per plant; OC, oil content; PH, plant height; SDI, Sclerotinia disease index; SPP, seeds per silique; SY, seed yield per unit area; TSW, thousand-seed weight; VDI, viral disease index; YPP, yield per plant.

## Data Availability

Data from this study can be found in the article.

## Supplementary Materials

The following supporting information can be downloaded at: https://www.mdpi.com/article/10.3390/plants15213337/s1, Table S1: QTL and association loci for growth duration, yield, quality, stress resistance, and agronomic traits in *Brassica napus*; Table S2: Candidate genes for growth duration, yield, quality, stress resistance, and agronomic traits in *Brassica napus*; Table S3: Phenotypic performance of early-maturing lines in the Chinese national regional trials (2021–2025).
